# Comparing Mouse and Rat Hippocampal Place Cell Activities and Firing Sequences in the Same Environments

**DOI:** 10.3389/fncel.2018.00332

**Published:** 2018-09-21

**Authors:** Xiang Mou, Jingheng Cheng, Yan S. W. Yu, Sara E. Kee, Daoyun Ji

**Affiliations:** ^1^Department of Molecular and Cellular Biology, Baylor College of Medicine, Houston, TX, United States; ^2^Department of Neuroscience, Baylor College of Medicine, Houston, TX, United States

**Keywords:** hippocampus, place cells, mouse, rat, theta sequences, replay

## Abstract

Hippocampal place cells are key to spatial representation and spatial memory processing. They fire at specific locations in a space (place fields) and fire in precise patterns during theta sequences and during ripple-associated replay events. These phenomena have been extensively studied in rats, but to a less extent in mice. The availability of versatile genetic manipulations gives mice an advantage for place cell studies. However, it is unknown how place fields and place cell sequences in the same environment differ between mice and rats. Here, we provide a quantitative comparison in place field properties, as well as theta sequences and replays, between rats and mice as they ran on the same novel track and as they rested afterwards. We found that place cells in mice display less spatial specificity with more but smaller place fields. Theta oscillations, theta phase precession and aspects of theta sequences in mice are similar as those in rats. The ripple-associated replay, however, is relatively rare during stopping on the novel track in mice. The replay is present during resting after the track running, but is weaker in mice than the replay in rats. Our results suggest that place cells in mice and rats are qualitatively similar, but with substantial quantitative differences.

## Introduction

Place cells are pyramidal neurons in the hippocampus that fire when an animal is physically at specific locations (place fields) in an environment (O'Keefe and Dostrovsky, 1971). Those place cells active in a given space are believed to represent an internal cognitive map of the space and are critically involved in spatial memory processing (O'Keefe and Nadal, 1978). Place cells have been extensively studied in rodents, especially after the development of high-density multi-channel recordings in freely moving animals (Wilson and McNaughton, 1993), and rapid progress has been made.

For example, place fields emerge quickly as an animal explores a novel environment (Wilson and McNaughton, 1993; Frank et al., 2004). Parameters of the newly emerged place fields, such as spatial specificity and place field size, may indicate how well the new environment is internally mapped. As the animal travels through place fields one by one along a trajectory, place cells fire one after another in a sequence. This firing sequence spans a behavioral time scale of a few to tens of seconds and forms a neural code of the trajectory (Harris et al., 2003). Interestingly, such behavioral firing sequences can also occur at a much smaller time scale of ~100 ms. First, when the animal runs along a trajectory, the local field potentials (LFPs) in the CA1 area of the hippocampus display prominent theta oscillations at 6–10 Hz. As the animal travels through a place field, the phases of the corresponding place cell's spikes occur earlier and earlier relative to each theta cycle, a phenomenon called theta phase precession (O'Keefe and Recce, 1993). Within a given theta cycle (~120 ms), those place cells active within the cycle display a sequence (theta sequence) similar to their behavioral firing sequence (Johnson and Redish, 2007). Second, when the animal stops on the track or rests/sleeps in a resting box, CA1 LFPs display high-frequency (100–250 Hz) oscillations in individual events called sharp-wave ripples (Buzsaki et al., 1992; Csicsvari et al., 2000). Within each ripple event (50–200 ms), those place cells active together often display a sequence (replay sequence) similar to the behavioral sequence on the track (Wilson and McNaughton, 1994; Lee and Wilson, 2002; Foster and Wilson, 2006; Diba and Buzsáki, 2007; Davidson et al., 2009; Karlsson and Frank, 2009). Both theta and replay sequences have been proposed to play important roles in spatial learning and memory (Ji and Wilson, 2007; Dupret et al., 2010; Carr et al., 2011; Pfeiffer and Foster, 2013; Wikenheiser and Redish, 2015; van de Ven et al., 2016; Zheng et al., 2016; Wu et al., 2017).

Progresses like these are mostly made from studies on place cells in rodents. In particular, place cell patterns such as theta and replay sequences are typically investigated in rats. Although many studies have examined place field properties in mice (e.g., McHugh et al., 1996, 2007; Kentros et al., 1998; Nakazawa et al., 2002; Cacucci et al., 2008), place cell sequences have been investigated only in a small number of mouse studies (Dragoi and Tonegawa, 2011; Cheng and Ji, 2013; Middleton and McHugh, 2016; Yamamoto and Tonegawa, 2017; Middleton et al., 2018). Given a wide range of available genetic mouse models, studying place cell patterns in mice may lead to better understanding of how spatial memory codes respond to circuit manipulations and how they are impaired in neurological and psychiatric disorders. However, place cells in rats and mice have not been directly compared in the same environment. It is unknown whether mouse place cells encode a space with the same precision as rat cells or whether place cell sequences are comparable between mice and rats. In this study, we aim to answer these questions by recording hippocampal place cells of rats and mice in the same novel environment and then comparing place field properties, as well as theta and replay sequences, between rats and mice.

## Results

We recorded hippocampal CA1 cells while 5 mice and 4 rats ran back and forth (two trajectories) for rewards on a rectangular 2-m track for 15–35 min and while they rested in a box afterwards for 20 min. The track was novel to the animals and they had never been exposed to the track before the recording day. The animals' behavior was variable on the novel track: The number of running laps per trajectory ranged between 6 and 20. But the median number of laps per trajectory was similar between mice (14 [7, 20]: median [25, 75%] values, same below unless specified; *N* = 10 trajectories) and rats (12 [7, 17.5], *N* = 8 trajectories; *P* = 0.68, Wilcoxon test). The overall running speed was higher in mice (14.7 [10.0, 18.4] cm/s; *N* = 10 trajectories), but did not reach statistical significance, compared to that in rats (11.9 [7.2, 14.2] cm/s; *N* = 8 trajectories; *P* = 0.36, Wilcoxon test). A total of 194 CA1 cells were recorded from mice and 181 from rats.

### Less spatial specificity of place cells in mice

We analyzed and compared place cells during track running between mice and rats. Since place cell properties may depend on animals' experience on the track, we focused our analysis on cell activities during the first 6 laps of each trajectory for all animals. In addition, we excluded the time periods when animals were at the reward sites or were stopping on the track, since CA1 cells can fire in a non-spatial manner in these periods. During the first 6 laps, the average running speed was significantly higher in mice (13.9 [12.3, 15.4] cm/s; *N* = 10 trajectories) than that in rats (11.3 [9.4, 13.1] cm/s; *N* = 8 trajectories; *P* = 0.043, Wilcoxon test). Furthermore, we only applied the analysis to a subset of cells with a spike sorting quality higher than a threshold (isolation distance >10). In the end, we obtained 50 putative pyramidal cells from mice and 39 from rats that were active within the first 6 laps of at least one trajectory (average firing rate >0.5 Hz and <5 Hz). For each of these two groups of cells, we computed its firing rate on each of the two running trajectories and found no significant difference between the groups in their median firing rates during the running (mice: 1.4 [0.79, 2.6] Hz, *N* = 100 cell × trajectory, meaning that each sample was a cell on a trajectory; same below. rats: 1.4 [0.81, 2.2] Hz, *N* = 78 cell × trajectory; *P* = 0.74, Wilcoxon test).

**Figure 1:**
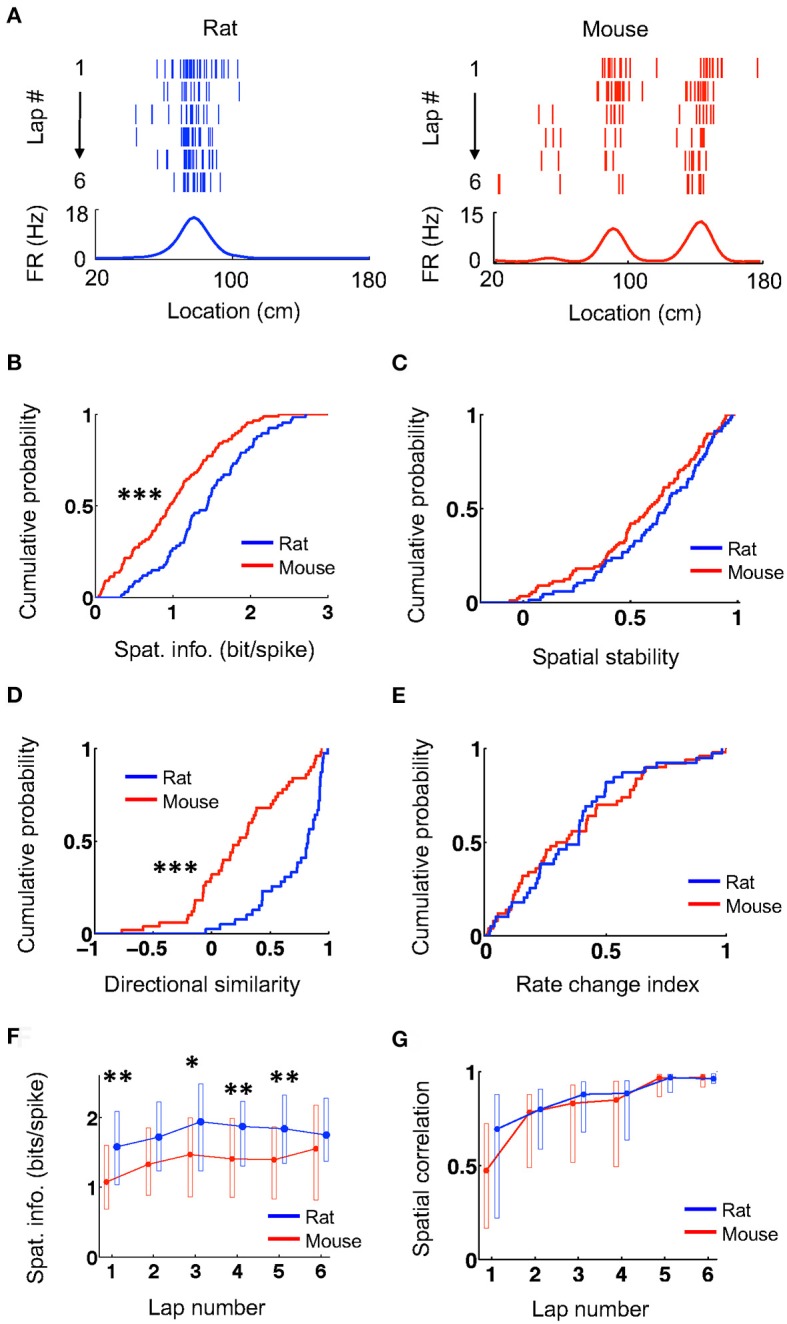
Mouse place cells had lower spatial information than rat ones. **(A)** Firing activities of an example rat place cell and an example mouse place cell during running on a novel track trajectory. For each panel, the top shows lap-by-lap spike raster during the first 6 laps on a linearized trajectory (running direction: from left to right). Each row is a lap and each tick is a spike. The bottom trace is the firing rate curve averaged over all 6 laps. **(B,C)** Cumulative distributions of spatial information **(B)** and spatial stability **(C)** for rat and mouse place cells on the novel track. ^***^P < 0.001. **(D,E)** Cumulative distributions of directional similarity **(D)** and rate change index **(E)** for rat and mouse place cells between the two running trajectories on the novel track. ^***^P < 0.001. **(F,G)** Lap-by-lap changes in spatial information **(F)** and in spatial correlation **(G)** between each lap' rate curve and the average rate curve of the last two laps. The plots represent median and [25, 75%] range values. ^**^P < 0.01, ^*^P < 0.05, Wilcoxon test; significance values adjusted for the multiple (6) comparisons.

We first examined the overall spatial firing activity of place cells on their active trajectories. Place cells in both rats and mice fired at specific locations and their firing locations were consistent from lap to lap, as shown by spike raster and firing rate curves on a trajectory (averaged firing rates vs. locations on the trajectory; Figure 1A). We quantified the spatial specificity of a place cell's firing rate curve by spatial information (Figure 1B), which measures the amount of information about an animal's location contained in a cell's firing activity (Skaggs et al., 1993). The spatial information of place cells in mice (0.97 [0.47 1.4] bit/spike, *N* = 88 cell × active trajectory) was significantly lower than that in rats (1.5 [0.98, 1.8], *N* = 67 cell × active trajectory; *P* = 4.0 × 10^−5^, Wilcoxon test). We then computed the firing rate curve of a place cell for each lap and quantified the consistency of its firing location cross laps by spatial stability (Figure 1C), which is the mean correlation among any two laps' rate curves. The spatial stability was comparable between mice (0.59 [0.39, 0.78], *N* = 88 cell × active trajectory) and rats (0.67 [0.46, 0.82], *N* = 67 cell × active trajectory; *P* = 0.14, Wilcoxon test). For each cell, we examined the directionality of its firing activity by a directional similarity, which was the correlation between the cell's firing rate curves on the two running trajectories of the track, and a rate change index, which measured the relative change of its peak rates on the two trajectories. We found that directional similarity was significantly lower in mice (0.24 [0.068, 0.56], *N* = 50 cells) than that in rats (0.82 [0.53, 0.92], *N* = 39 cells; *P* = 2.0 × 10^−7^, Wilcoxon test; Figure 1D), but rate change index was similar between mice (0.31 [0.13, 0.60], *N* = 50 cells) and rats (0.39 [0.19, 0.48], *N* = 39 cells; *P* = 0.99, Wilcoxon test; Figure 1E). We also examined the lap-by-lap dynamics in a place cell's firing. The spatial information in mice started significantly lower than that in rats and appeared to stay lower throughout most of the laps (Figure 1F). Finally, the correlation between the firing rate curve in each lap and the average curve of the final two laps (lap 5 and 6) shows that firing locations of place cells in mice and rats were stabilized lap by lap in a similar manner (Figure 1G). These results indicate that the firing activities of place cells in mice had lower spatial specificity and lower directional similarity (thus higher directionality) on the novel track, but otherwise were comparable to those of rats in terms of their spatial stability and firing dynamics.

**Figure 2:**
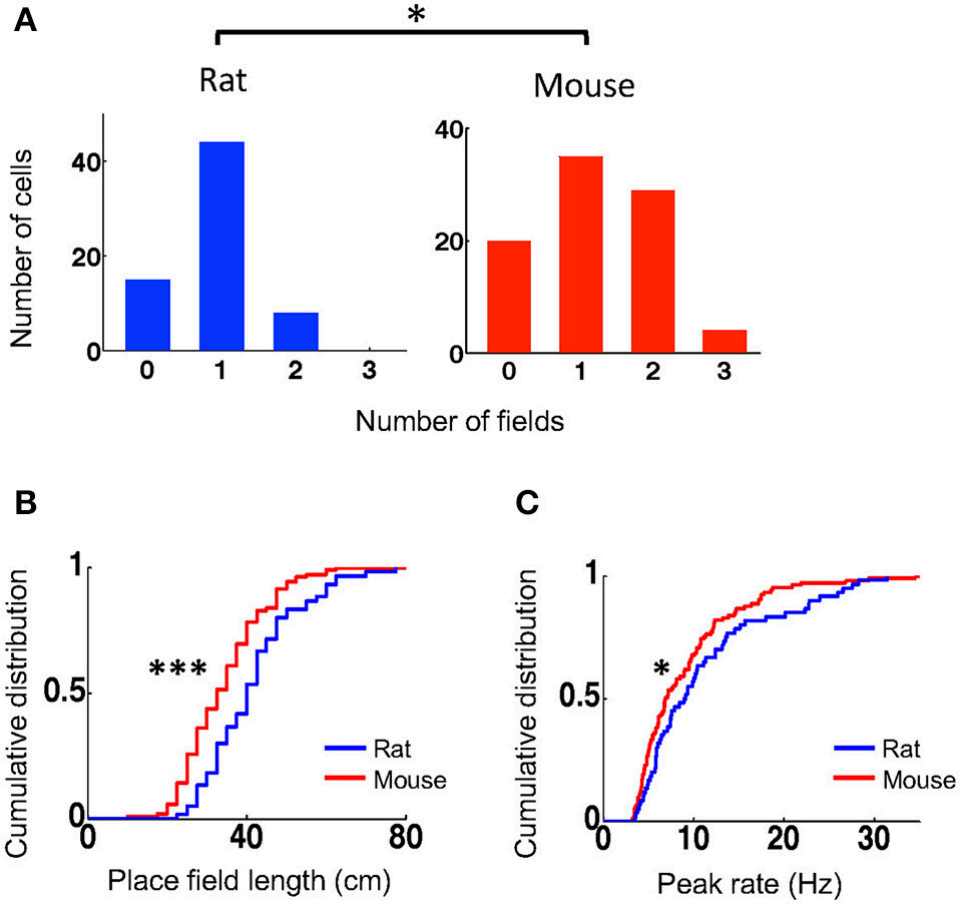
Mouse place cells had more place fields with shorter lengths and lower peak rates than rat place cells. **(A)** Histograms for rat and mouse place cells with different number of place fields on an active trajectory. ^*^P < 0.05. **(B,C)** Cumulative distributions of place field length **(B)** and peak firing rate **(C)** for rat and mouse place cells on the novel track. ^***^P < 0.001, ^*^P < 0.05.

To understand what led to the lower spatial specificity of place cells in mice, we compared their place field properties to those of rat place cells. We identified each place field of a place cell and then quantified the number of place fields per active trajectory, place field length, and within-field peak firing rate. Place cells in mice on average had more place fields per active trajectory (mean ± se: 1.2 ± 0.09, *N* = 88 cell × active trajectory) than those in rats (0.90 ± 0.07, *N* = 67 cell × active trajectory; *P* = 0.014, Student's *t*-test; Figure 2A). A small percentage of cells in rats (12%) fired at more than one place fields on a trajectory, whereas 36% of place cells in mice (*P* = 0.00057, binomial test) did so. Place fields in mice had significantly shorter length (33 [25, 40] cm, *N* = 105 fields) that those in rats (40 [33, 48] cm, *N* = 60 fields; *P* = 9.2 × 10^−5^, Wilcoxon test; Figure 2B). Place cells in mice also had lower peak firing rate within their place fields (6.9 [4.8, 11] Hz, *N* = 105 fields), compared to those in rats (9.0 [5.8, 14] Hz, *N* = 60 fields; *P* = 0.049, Wilcoxon test; Figure 2C). The place field analysis thus indicates that place cells in mice represented the same novel track with more, but smaller and weaker, place fields than place cells in rats did.

### Similar theta oscillations and phase precession between mice and rats

**Figure 3:**
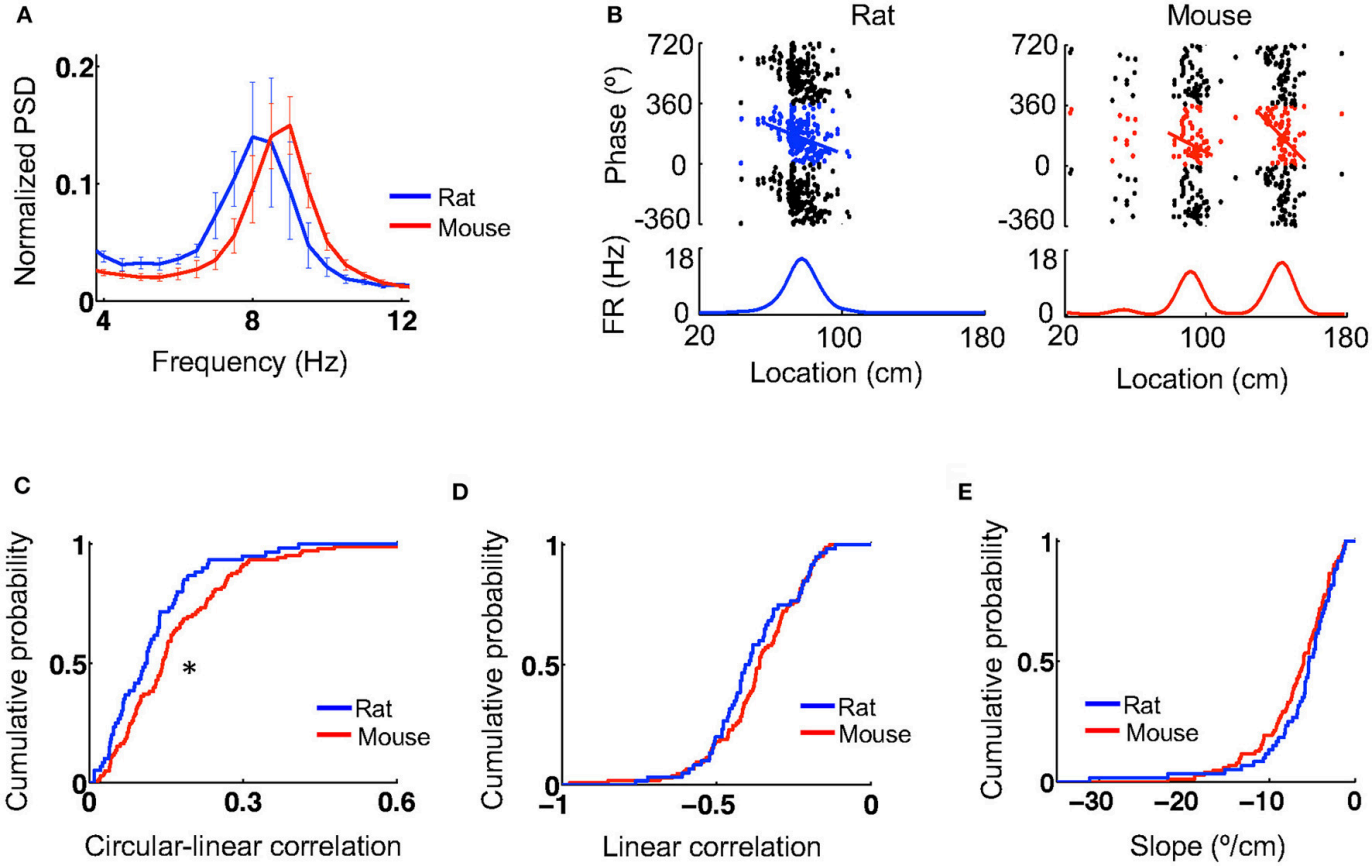
Theta oscillations and theta phase precession were similar between mice and rats. **(A)** Average (mean and s.e.) power spectral densities (PSDs) of mouse and rat CA1 LFPs. The PSDs were normalized by the total power within [2,400] Hz. **(B)** Theta phase precession of the same example cells in Figure 1A. In each panel, the top shows spike phases plotted against their locations on the track. The same spikes were plotted for 3 cycles of circular phases. Line: optimal linear regression between spike phases and locations. The bottom shows the firing rate curve of the cell. **(C–E)** Cumulative distributions of circulation-linear correlation **(C)**, optimal linear correlation **(D)**, and associated slope **(E)** between spike phases and spike locations for rat and mouse place cells on the novel track. ^*^P < 0.05.

During active behavior such as track running, CA1 LFPs display prominent theta oscillations at 6–10 Hz. We next examined theta characteristics and theta phase precession of place cells when animals ran on the novel track. Power spectral density (PSD) analysis reveals a prominent peak in the theta band in the CA1 LFPs of both mice and rats (Figure 3A). The total power within the theta range of 6–10 Hz (normalized by the total power within [2, 400] Hz) was similar between mice (0.31 [0.26, 0.42], *N* = 5 mice) and rats (0.38 [0.23, 0.47], *N* = 4 rats; *P* = 1.0, Wilcoxon test). The peak theta frequency in mice (9 [8.4, 9] Hz, *N* = 5 mice) appeared to be slightly higher than that in rats (7.8 [7.5, 8.3], *N* = 4 rats), but did not reach the level of significance (*P* = 0.079, Wilcoxon test). The peak theta power appeared to be similar between mice (0.16 [0.15, 0.21], *N* = 5 mice) and rats (0.18 [0.097, 0.23], *N* = 4 rats; *P* = 0.090, Wilcoxon test).

Plotting spike theta phases vs. spike locations within place fields displays prominent theta phase precession in both rats and mice (Figure 3B). We quantified theta phase precession by a circular-linear correlation between spike phases and spike locations, as well as a best-fit linear regression and its associated slope. The median circular-linear correlation was modestly (27%) higher for place fields in mice (0.14 [0.082, 0.23], *N* = 105 fields) than those in rats (0.11 [0.054, 0.16], *N* = 60 fields; *P* = 0.0072, Wilcoxon test; Figure 3C), but the median linear correlation associated with the optimal linear regression was similar between mice (−0.36 [−0.44, −0.26], *N* = 105 fields) and rats (−0.40 [−0.48, −0.28], *N* = 60 fields; *P* = 0.21, Wilcoxon test; Figure 3D). In addition, there was also no significant difference in the linear regression slope between mice (−6.0 [−9.1, −3.8]°/cm, *N* = 105 fields) and rats (−5.4 [−7.6, −3.3]°/cm, *N* = 60 fields; *P* = 0.11, Wilcoxon test; Figure 3E). Our analysis thus indicates that theta oscillations and theta phase precession in mice were largely similar to those in rats, although minor differences were found.

### Similar bayesian decoding of track locations

**Figure 4:**
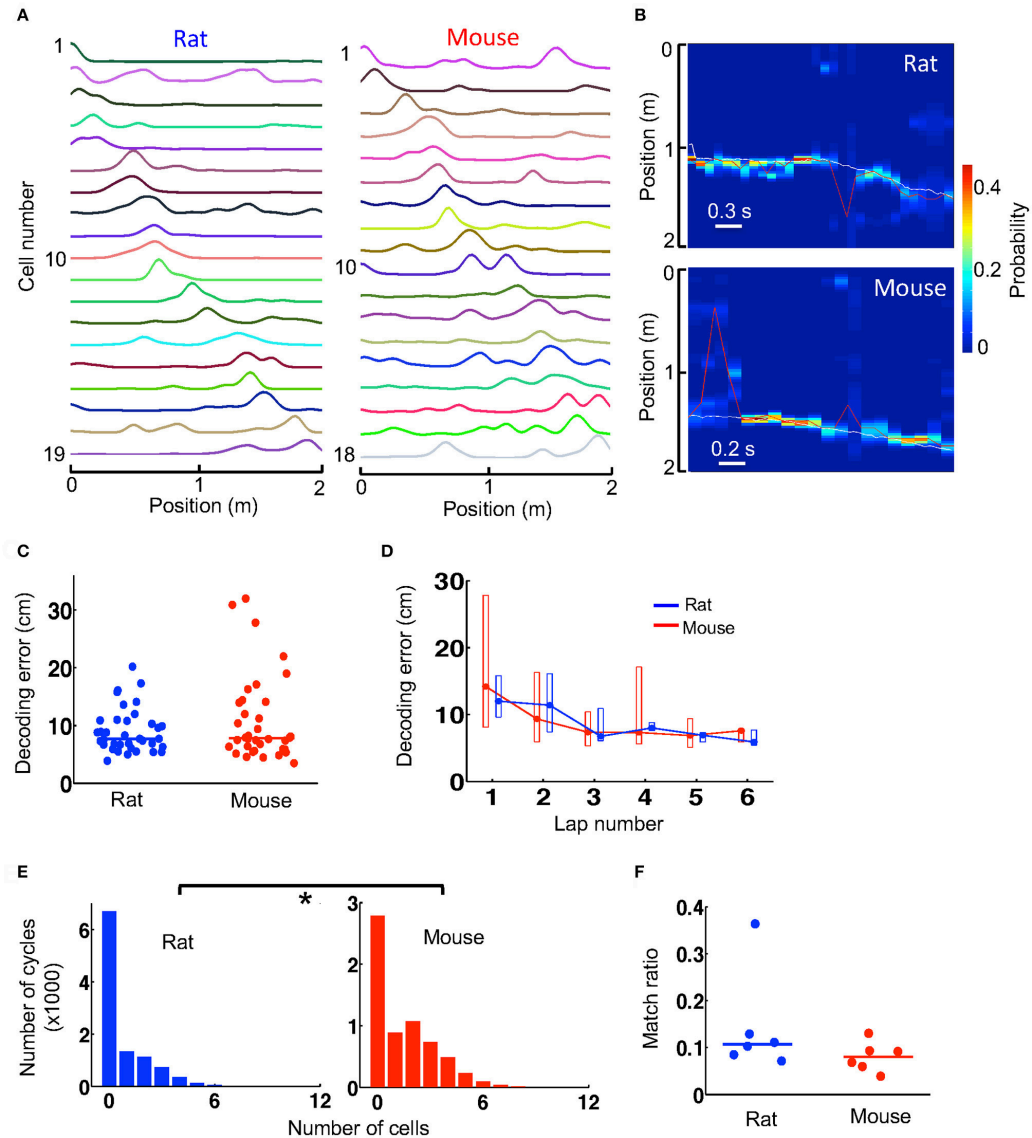
Spatial locations during track running were decoded with similar accuracy from mouse and rat place cell activities. **(A)** An example rat template and an example mouse template on a trajectory. Each line is the firing rate curve of a place cell. **(B)** Decoded probability of each position at each time point (100 ms) for a track running period of a rat and for a running period of a mouse. The decoding was based on the two templates in **(A)**. Color represents decoded probability. Red line: decoded position (peak probability location) at each time point. White line: animal's actual position. Note the close match between the decoded (red) and actual (white) positions at most of the time points. **(C)** Decoding error of every lap for all rats and mice during running of the novel track. Lines: median values. **(D)** Lap-by-lap decoding error (median and [25, 75%] range values). **(E)** Histograms of rat and mouse theta cycles with different number of active template cells. ^*^P < 0.05. **(F)** Match ratio of the number of theta cycles within which decoded positions matched a portion of the track over all candidate theta cycles. Each dot is a trajectory of an animal.

Since firing activities of place cells contain information about an animal's spatial location, they can be used to decode the animal's locations in a Bayesian scheme. Given the lower spatial information of place cells in mice, we asked whether spatial locations of mice could be decoded with similar accuracy as those decoded in rats. Because Bayesian decoding can use not well-sorted cells (Kloosterman et al., 2014), for this analysis we considered all the recorded cells that were active on a trajectory, which varied from 5 to 34 in number, and built a template from their firing rate curves on the trajectory, all averaged over the first 6 laps. However, for a fair comparison, it was necessary to equalize the number of cells in mouse and rat templates. Therefore, we removed templates with too few active cells (<12) and randomly downsampled the cells in those templates with too many cells (>20). In the end, we obtained 6 templates from 3 mice and 6 templates from 3 rats with similar number of cells (median and range numbers: rat−14 [12, 18], mice−14 [12, 20]). One mouse and one rat template is shown in Figure 4A.

We decoded the animal's location on a trajectory at every 100 ms during the first 6 running laps, based on the firing activities of all place cells contained in the trajectory's template. The decoded locations during two time windows (decoded from the two templates in Figure 4A), as well as the animals' actual locations, are shown in Figure 4B. We quantified the decoding error at every time point by the absolute difference between the decoded location and the animal's actual location and used the median error of all time points in a lap as a measure of decoding accuracy. We found that the median decoding errors were similar between mice (7.8 [5.9, 14] cm, *N* = 36 laps in 3 mice) and rats (7.7 [6.5, 10.9] cm, *N* = 36 laps in 3 rats; *P* = 0.82, Wilcoxon test; Figure 4C). The result suggests that the animals' locations could be decoded equally well in mice as in rats. We also examined the lap-by-lap dynamics in the decoding errors. The errors started relatively high and dropped in later laps both in mice and rats and there were no significant differences in decoding error in any of the laps (Figure 4D).

Bayesian decoding can be performed at a finer time scale of 10 ms within each theta cycle of the running to identify theta sequences. With the templates we had, the number of template cells active within a theta cycle was low in general (Figure 4E). Nevertheless, we found that the median number of active cells per cycle in mice (1 [0, 3], *N* = 6,347 cycles) was more than that in rats (0 [0, 1], *N* = 10,458 cycles; *P* < 1.0 × 10^−6^, Wilcoxon test; Figure 4E). We considered those theta cycles with 4 or more active cells as candidate cycles. For each candidate cycle on a trajectory, we performed Bayesian decoding at every 10 ms from the trajectory's template and determined whether the sequence of the decoded locations within the cycle matched a portion of the trajectory. We found that the match ratio of the number of matched cycles to the total number of candidate cycles was similar between mice (0.080 [0.060, 0.093], *N* = 6 templates) and rats (0.11 [0.085, 0.13], *N* = 6 templates; *P* = 0.18, Wilcoxon test; Figure 4F). The result suggests that theta sequences were similar between mice and rats.

### Lower ripple frequency in mice

**Figure 5:**
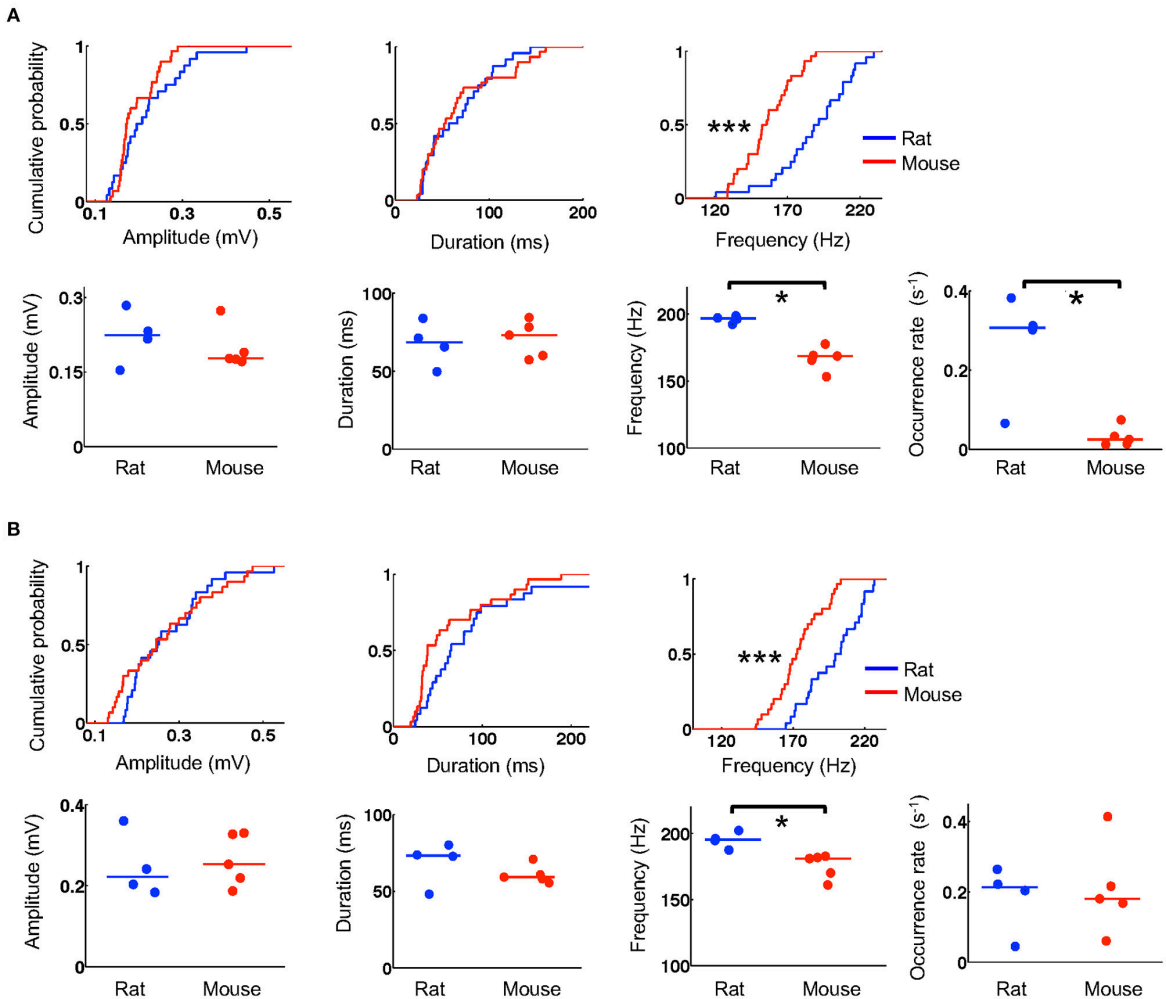
Mouse ripples had lower frequencies. **(A)** Quantifications of ripple parameters (amplitude, duration, frequency, occurrence rate) during stopping on the novel track. For each column, the top shows the cumulative distribution of a parameter computed from individual ripples sampled equally from all animals in the rat and mouse group (*N* = 24 ripples from 4 rats, 30 ripples from 5 mice; 6 ripples per animal). The bottom shows the parameter's values for individual animals averaged from all ripples detected on the track. Lines: median values. ^*^P < 0.05, ^***^P < 0.001. **(B)** Same as in **(A)**, but for ripples during resting in a box after the track running.

We next examined whether the templates during running were replayed within ripple events during stopping on the track and during resting in a box after the running. We first identified and quantified individual ripple events by their amplitude, duration, and frequency and then compared these parameters between mice and rats. The number of ripples was large in some animals and varied across animals (6–480 on the novel track, 39–530 during resting). To avoid detecting small statistically significant differences that might be biologically irrelevant (due to large number of samples) and to ensure that the results were not biased by one or two animals, we randomly re-sampled the data to 6 ripples per animal per session and performed the comparisons between these samples from 5 mice and 4 rats. During stopping on the track, we found that ripples in mice and rats had similar amplitudes (mice: 0.17 [0.16, 0.23] mV, *N* = 30 ripples; rats: 0.20 [0.17, 0.27] mV, *N* = 24 ripples; *P* = 0.24, Wilcoxon test) and duration (mice: 53 [35, 92] ms, *N* = 30 ripples; rats: 62 [34, 92] ms, *N* = 24 ripples; *P* = 0.85, Wilcoxon test), but ripples in mice had lower frequencies than those in rats (mice: 154 [143, 170] Hz, *N* = 30 ripples; rats: 190 [174, 208] Hz, *N* = 24 ripples; *P* = 1.2 × 10^−5^, Wilcoxon test; Figure 5A). During resting, ripples in mice and rats again had comparable amplitudes (mice: 0.25 [0.17, 0.34] mV, *N* = 30 ripples; rats: 0.25 [0.20, 0.33] mV, *N* = 24 ripples; *P* = 0.62, Wilcoxon test) and duration (mice: 38 [32, 87] ms, *N* = 30 ripples; rats: 65 [43, 96] ms, *N* = 24 ripples; *P* = 0.068, Wilcoxon test), but significantly lower frequencies (mice: 172 [162, 185] Hz, *N* = 30 ripples; rats: 201 [182, 217] Hz, *N* = 24 ripples; *P* = 4.6 × 10^−5^, Wilcoxon test; Figure 5B).

To examine whether the detected differences were representative of all ripples in majority of the animals, we computed an average value for each ripple parameter over all identified ripples in an animal. We found that the observed differences in ripple frequency between rats and mice were reflected by the average values of individual animals both on the novel track (Figure 5A) and during resting (Figure 5B). Specifically, during stopping on the track, average amplitudes were similar between mice and rats (mice: 0.18 [0.17, 0.21] mV, *N* = 5 mice; rats: 0.22 [0.19, 0.26] mV, *N* = 4 rats; *P* = 0.56, Wilcoxon test). The average durations were also similar (mice: 73 [59, 80] ms, *N* = 5 mice; rats: 68 [58, 77] ms, *N* = 4 rats; *P* = 0.73, Wilcoxon test), but the average frequencies were significantly lower in mice than those in rats (mice: 169 [162, 171] Hz, *N* = 5 mice; rats: 197 [194, 198] Hz, *N* = 4 rats; *P* = 0.016, Wilcoxon test; Figure 5A). During resting, average amplitudes in mice and rats again were similar (mice: 0.25 [0.21, 0.33] mV, *N* = 5 mice; rats: 0.22 [0.19, 0.30] mV, *N* = 4 rats; *P* = 0.73, Wilcoxon test) and so were the average durations (mice: 59 [57, 63] ms, *N* = 5 mice; rats: 73 [60, 77] ms, *N* = 4 rats; *P* = 0.29, Wilcoxon test), but average frequencies were again significantly lower in mice (mice: 181 [168, 182] Hz, *N* = 5 mice; rats: 195 [191, 199] Hz, *N* = 4 rats; *P* = 0.016, Wilcoxon test; Figure 5B). Furthermore, we computed an occurrence rate of ripples in a session for each animal. We found that ripple occurrence rate in mice was significantly lower than that in rats during the stopping on the novel track (mice: 0.025 [0.014, 0.043] s^−1^, *N* = 5 mice; rats: 0.31 [0.18, 0.35] s^−1^, *N* = 4 rats; *P* = 0.032, Wilcoxon test), but not during resting afterwards (mice: 0.18 [0.14, 0.27] s^−1^, *N* = 5 mice; rats: 0.21 [0.12, 0.24] s^−1^, *N* = 4 rats; *P* = 0.90, Wilcoxon test). These quantifications show that ripples in mice had lower frequencies and appeared to occur less frequently during stopping on the novel track than ripples in rats.

### Weaker replay during stopping and resting in mice

**Figure 6:**
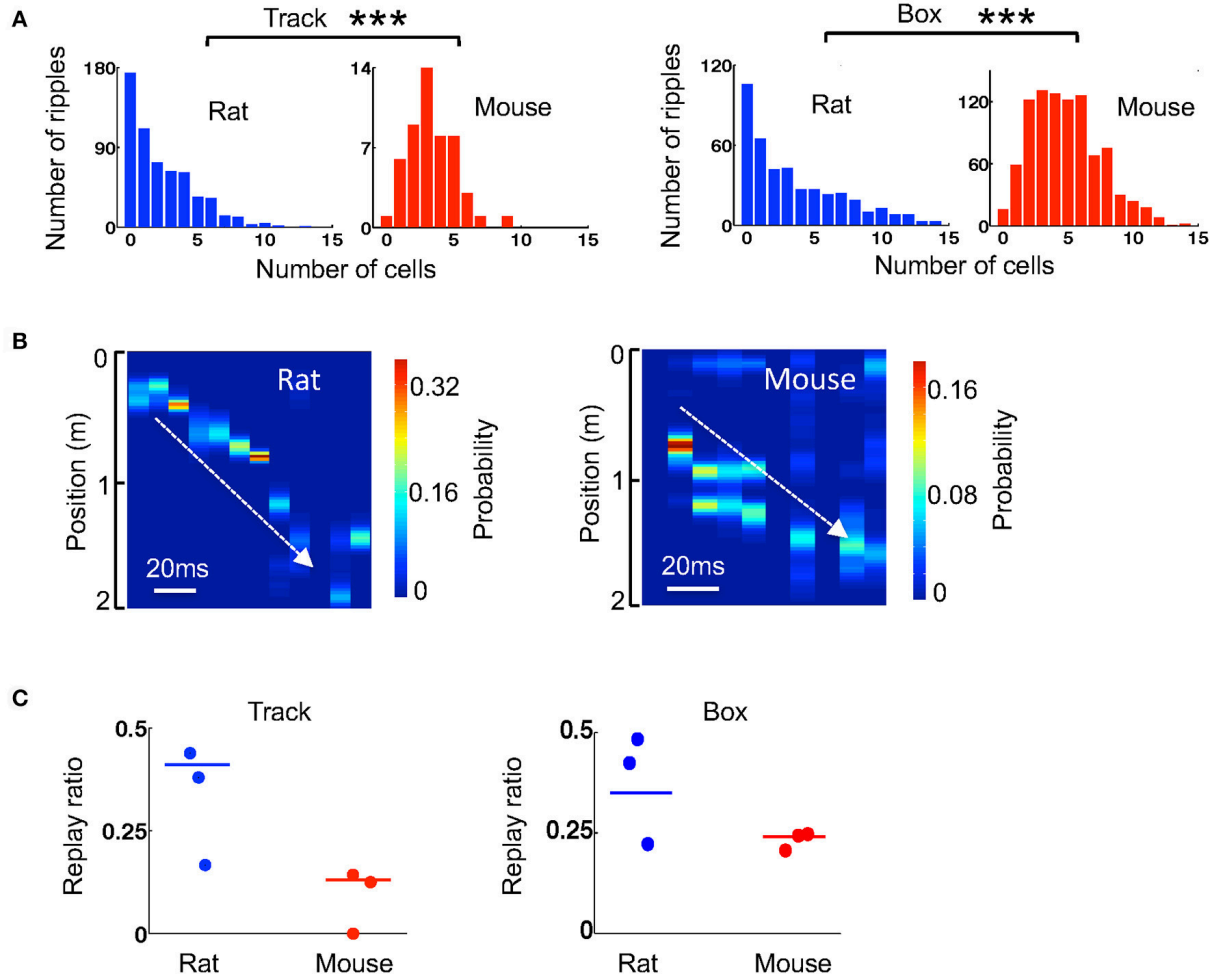
Ripple-associated replay was relatively weaker in mice than that in rats. **(A)** Histograms of rat and mouse ripples with different number of active template cells during stopping on the track and during resting in the box. ^***^P < 0.001. **(B)** An example replay event in a rat and one in a mouse identified by Bayesian decoding. Color represents the decoded probability of each position at each time point (10 ms) of a ripple. The decoding was based on the two templates in Figure 4A. Note that the decoded peak locations replayed a part of a trajectory on the track (white line) in both examples. **(C)** Replay ratio of the number of replay events over all candidate ripples during stopping on the track and during resting in a box for every animal. Each dot indicates an animal. Solid lines: group replay ratios (number of all replay events over all candidate ripples combined from all animals of a group).

We next examined how the template sequences during active track running in the 3 mice and 3 rats were replayed within ripples. Since replay may depend on time duration within or after the running experience (Lee and Wilson, 2002; Ji and Wilson, 2007), we quantified the replay during stopping on the track within first 6 running laps and during resting in a box within first 20 min after the running. We first quantified how many template cells were active together in a ripple event. Because cells in a ripple could replay one of the two templates on the track, we averaged the numbers over the two templates. For ripples during stopping on the track, the median number of coactive cells per ripple was higher in mice (3.5 [2.5, 4.9], *N* = 51 ripples) than that in rats (2.0 [0.5, 4.0], *N* = 586 ripples; *P* = 2.2 × 10^−5^, Wilcoxon test; Figure 6A). For the ripples during resting in a box after running, the finding was similar (mice: 5.0 [3.0, 6.5], *N* = 930 ripples; rats: 2.5 [0.5, 6.0], *N* = 421 ripples; *P* = 2.3 × 10^−21^, Wilcoxon test; Figure 6A). Therefore, consistent with activities within theta cycles, there were more cells coactive within ripples in mice than in rats.

We considered those ripples with at least 4 active cells in any of the two templates as candidate ripples. For each candidate ripple, we used the Bayesian decoding method to identify whether it replayed any of the two templates during track running (Figure 6B). If so, we counted it as a replay event. We then computed a replay ratio, which was the ratio of the number of replay events to the total number of candidate ripples. Since the ripple occurrence rate, and therefore the number of candidate ripples, were low in mice during stopping on the track, we combined the ripples from all 3 mice and those from all 3 rats and compared the ratio between mice and rats. During the stopping on the track, we found only a few replay events (*N* = 4 replay events out of 33 candidates) among the 3 mice and the replay ratio was significantly lower than that in rats (mice: 13%; rats: 41%; *P* = 0.0020, binomial test). However, during resting in a box after the track running, although ripples and therefore replay events were frequently observed in mice (*N* = 171 replay events out of 722 candidates), the replay ratio in mice remained significantly lower than that in rats (mice: 24%; rats: 35%; *P* = 0.0014, binomial test). The replay ratios in individual animals varied, but in general were consistent with the group averages (Figure 6C). Therefore, our data suggest that ripple-associated replay was relatively weak during stopping on the track, but was present during resting, in our mice. In both cases, the replay appeared to be less prominent in mice than in rats.

## Discussion

By comparing place cell activities and their firing patterns in the same environment, we identified similarities and differences between mouse and rat place cells. Mouse place cells are qualitatively similar to rat ones. Their place fields develop quickly on a novel track, they display similar theta phase precession and similar theta sequences as rat place cells, and their sequences are replayed in ripple events. Yet major quantitative differences exist. Mouse place cells are less spatially specific and more directional with more, smaller place fields on the novel track. They are more active in theta cycles and ripple events than rat place cells. Interestingly, ripple and replay events are rare during the first few laps on the novel track in mice and the replay in mice is weaker during resting than that in rats. These results suggest that mouse and rat place cells represent the same environment in a similar manner. However, the differences identified here, especially those in ripples and replay, may lead to different spatial learning and memory processing in the same environment between mice and rats.

Place cells have been extensively studied in rodents. The seemingly similar nature of location-specific firing in both rat and mouse place cells indicates that spatial representation schemes are similar in rats and mice. A previous review on existing literature found that mouse place cells have less information content and have less stable place fields than rat place cells across sessions and days (Hok et al., 2016). However, to our knowledge, the emergence of place fields in the same novel environment had not been directly compared between rats and mice. For this purpose, we intentionally used a 2-m long novel track in this study, which is longer than the apparatus in a typical mouse study but shorter than that in a typical rat study. We found that rat and mouse place fields emerge in novel environments with similar dynamics and are similarly stable in the shorter time scale of laps. Our study also verified that mouse place cells have lower spatial information than rat place cells in the same environment. Our place field analysis also suggests that mouse place cells tend to have multiple, smaller place fields in novel environments. Given the much smaller body size of a mouse (~7 cm) than that of a rat (~19 cm), the smaller place fields in mice are expected, although the ~20% difference in place field size (median: 33 cm in mice, 40 cm in rats) falls short of proportion to their body size difference. Given that the track was relatively long to mice, it is also expected that multiple fields tend to occur more often in mice than in rats (Kjelstrup et al., 2008; Davidson et al., 2009; Rich et al., 2014). The relatively long track to mice is probably also related to another finding in our study: Mouse place fields appear to be more directional. Longer tracks make individual locations more distinctive and make animals easier to recognize which direction they are moving. Nevertheless, direct comparisons of mice and rats performing the same task in the same novel environment allowed us to reveal quantitative differences of place cells under same experimental conditions.

At the level of neuronal populations, studies on place cell firing patterns and sequences are important to our current understanding of memory processing. Such studies are mostly done in rats. Similar ones in mice have emerged only recently (Dragoi and Tonegawa, 2011; Cheng and Ji, 2013; Middleton and McHugh, 2016; Yamamoto and Tonegawa, 2017; Middleton et al., 2018). No previous studies have compared place cell sequences in the same environments between rats and mice. Our data here show that place cell ensembles can be used to decode the same space with similar accuracy between rats and mice. Theta phase precession and theta sequence quality (measured by match ratio) are also similar between rats and mice. Our data thus suggest that, despite the quantitative differences in spatial information and place field parameters, spatial representations at the neuronal ensemble level are similar between rats and mice. However, one key difference we found is that awake replay during ripples at least during the first few laps on the track is rare and replay during resting after the track running appears weaker in mice. Because ripples and ripple-associated replay are important for memory processing (Girardeau et al., 2009; Ego-Stengel and Wilson, 2010; Jadhav et al., 2012) and place field stability (van de Ven et al., 2016; Roux et al., 2017; but see Kovacs et al., 2016), our finding may explain why place fields in mice appear less stable between sessions and across days and why mice and rats perform differently in same environments (Hok et al., 2016).

However, we need to be cautious on the interpretation of the differences in place cells and replay between mice and rats. One important issue often hard to disentangle is whether they are simply caused by differences in behavioral parameters. In our study, we tried our best to conduct the comparison between mice and rats at similar time points of the experience (first 6 laps on the novel track, 20 min resting following the track running) and with similar qualities of place cells and templates. Nevertheless, the behaviors of our mice and rats were not identical. Our mice ran faster and were therefore more active than rats on the novel track. This seemingly hyperactive behavior may explain the decreased number of ripples and awake replay on the track. Alternatively, the lack of replay may indicate that our mice were just busy moving around in the environment and did not stop and “think” much to learn about the environment. In addition, although our mice, and perhaps mice in general, are hyperactive, not all mice are hyperactive in all behavior. It is possible that in some behavioral tasks or after more running laps/experience mice may pause more often and ripples and therefore awake replay may increase (Yamamoto and Tonegawa, 2017; Middleton et al., 2018). In any case, a true cognitive basis of place cell differences between rats and mice need further investigation.

The hyperactivity issue may indicate just one possible limitation of using mice for the study of place cells and place cell patterns. Another limitation is obviously the number of simultaneously recorded neurons. Whereas hundreds of neurons can be simultaneously recorded from a rat (Pfeiffer and Foster, 2013), the number is about an order of magnitude less in freely moving mice. The smaller number of neurons limits the power of place cell pattern analysis, although new technological developments may solve the problem (Jun et al., 2017). Furthermore, mice are generally harder to handle and train in complex tasks. However, there is an advantage of mice that rats cannot match at least at the moment. There is a wide range of transgenic mouse models that allow circuit manipulations and the study of many neurological and psychiatric disorders. Because place cells and theta sequences are qualitatively similar between mice and rats and replay still occurs in mice, studying place cells in mouse models may advance our understanding of how place cell patterns are produced by the hippocampal circuitry and how they are altered in various brain disorders (Cheng and Ji, 2013; Suh et al., 2013; Middleton et al., 2018).

In summary, our study reveals key similarities and differences in hippocampal place cells and place cell patterns between mice and rats in the same environments. The differences suggest that mice may learn about environments and process spatial memories differently from rats. The similarities suggest that basic spatial representations are comparable between rats and mice and transgenic mouse models may be invaluable for understanding the functions of place cell patterns and their role in brain disorders.

## Methods

### Animals and behavioral procedure

Four rats and5 mice, all adults at 3–9 months old, were used in this study. All rats were male, Long–Evans rats. Two of the mice were female F1 offsprings between a FVB/N strain that carried a *Mecp2*^±^ gene and a pure 129S6 strain (Guy et al., 2001). Three other mice were male F1 offsprings between a FVB strain that carried a human tau gene with the P301L mutation and a 129S6 strain that carried the tTA transgene (Santacruz et al., 2005). However, all mice in this study were wild type animals without the expression of any of the transgenes. All animals were implanted with a tetrode drive in a surgery (see below). Animals were mildly food- or water-restricted with weight ≥85% of *ad libitum* level and trained to run back and forth on a familiar track for food rewards. Recording started when animals did the same task first day on a novel task (see below) in a novel room. Different animals were recorded on different days. All research and animal care procedures followed the recommendations in the “Guide for the Care and Use of Laboratory Animals” of the National Institute of Health and were approved by the Baylor College of Medicine Institutional Animal Care and Use Committee.

### Surgery

A tetrode drive was surgically implanted to every animal. The mouse drive contained 8 tetrodes aimed at the CA1 area of the hippocampus and a reference electrode to the white matter above the hippocampus. The rat drive contained 16 tetrodes to the CA1 and a reference. The animal was anesthetized with 0.5–3% isoflurane and mounted on a stereotaxic device. The coordinates of the implantation site for CA1 was antereoposterior (AP) 2.0 mm and mediolateral (ML) 1.5 mm from the Bregma in mice, and AP 3.8 mm, ML 2.5 mm in rats. The tetrode drive was mounted to the skull using stainless anchoring bone screws and dental cement.

### Tetrode recording and behavioral task

Tetrode recording was conducted as previously described (Cheng and Ji, 2013; Mou and Ji, 2016, 2018). In 2–4 weeks after the surgery, tetrodes in every animal were slowly moved down to the CA1 pyramidal layer, which was identified by sharp-wave ripples in LFPs and bursting spikes during resting. Once spike clusters were visually stable for at least 2 days, recording of LFPs and spikes was made by a Neuralynx Digital Lynx system. LFPs were filtered between 0.5 Hz−1 kHz and sampled at 2 kHz. Spikes were identified by a threshold of 50–70 μV after filtering between 600 Hz and 9 kHz and sampled at 32 kHz. The animal's positions, sampled at 33 Hz, were tracked by two diodes mounted to the tetrode drive and recorded by an overhead camera.

The recording was made while the animal was running back and forth (two trajectories) for food or water rewards on a novel track and while resting in a box. The track was ~2 m long and rectangle in shape with two reward sites at one corner (Cheng and Ji, 2013). The recording lasted for 15–35 min on the novel track and 20–60 min in the resting box. However, the spike data were analyzed only within the first 6 running laps (on each trajectory) on the track and within the first 20 min of resting in the box.

### Histology

After the recording, animals were sacrificed with pentobarbital overdose (50 mg/kg). A 30 μA current was passed to each tetrode for ~10 s to produce a small lesion at each recoding site. The brain was dissected, fixed with 10% formalin or cryoprotected in 30% sucrose, sectioned at 50–100 μm thickness, and stained with 0.2% Cresyl violet. Recording sites at the CA1 pyramidal layer were identified by the lesions.

### Data analysis

Single units (spikes from single neurons) were sorted offline using a manual clustering program xclust (M. Wilson, MIT). Cluster quality was assessed using isolation distance (Schmitzer-Torbert et al., 2005). Only putative pyramidal neurons active on at least one of the two trajectories, identified as overall firing rate on the trajectory between 0.5 and 5 Hz, were included in the analysis. For quantitative comparisons on place cell properties, only those cells with isolation distance >10 were included. For theta and replay decoding analysis, all sorted cells active on a trajectory were considered. However, the cells on some of the rat and mouse trajectories were randomly selected to equalize the number of cells per trajectory between rats and mice. Results were presented as median and [25, 75]% range values, unless described otherwise. Statistical comparisons were made by the Wilcoxon test, the binomial test, or the Student's *t*-test.

#### Place cell properties and place field analysis

The two trajectories of the novel track were linearized. For each cell active on a trajectory, its firing rate curve was computed as the average firing rate among the first 6 laps at each spatial bin of the linearized trajectory with a bin size of 2.5 cm. The start and end portions (~20 cm) of each trajectory were excluded from the rate curve computation. The time periods when animals stopped (speed <5 cm/s for at least 0.5 s) in the middle of the trajectory were also excluded. Spatial information of the rate curve in bit/spike was computed from the standard formula (Skaggs et al., 1993). We computed spatial information for each cell separately on each of its two possible active trajectories (cell × active trajectory). Directional similarity was computed as the Pearson correlation between a cell's two firing rate curves on the two running trajectories of the track. A cell's rate change index between the two rate curves was defined from their peak rates as their absolute difference divided by their sum. We also computed the firing rate curve for each of the first 6 laps. Spatial stability was the average correlation value among all pairs of laps' rate curves. Spatial information was computed at each lap for every cell to obtain the lap-by-lap dynamics. The correlation between a lap's rate curve and the average of the last two laps (5, 6) was computed for every cell to characterize the stabilization of novel place fields. Place fields were identified by a peak rate ≥3 Hz and boundaries were determined by a threshold of 10% of peak rate. Only place fields with a minimum of 5 cm were considered. Because here we applied a threshold of peak rate of 3 Hz to our identification of place fields, not all cells active on a trajectory had identifiable place fields, which was observed more often in the first a few laps of a novel track since place fields were premature (Frank et al., 2004).

#### Theta power and theta phase precession

For each animal, we analyzed LFPs recorded by one channel of a tetrode at the CA1 pyramidal cell layer, identified by the presence of prominent ripples during resting and at least 3 single units. We computed the power spectral density (PSD) of raw LFPs during active running on the novel track, using a multitaper method in Matlab. PSDs were then normalized by the total power between 2 and 400 Hz. Theta power was the integration of PSDs within 6–10 Hz. For theta phase precession analysis, each raw LFP was filtered within 6–12 Hz and its peak times were identified. Theta phase of each spike of an active place cell was computed from its spike time relative to the closest peak time (0°/360°). The spike phases and spike locations of all spikes within a place field were used to compute a circular-linear correlation as previously described (Ravassard et al., 2013). We also computed an optimal linear correlation between spike phases and spike locations (O'Keefe and Recce, 1993). In this case, the spike phases of all spikes were shifted 1° by 1°. A linear regression between spike phases and spike locations were computed at every shift and the corresponding correlation and regression slope were obtained. The optimal linear correlation was the one with the maximum absolute correlation value. Its associated slope was used to measure how fast theta phases processed within a place field.

#### Ripple analysis

Sharp-wave ripples were detected from LFPs filtered within [100, 250] Hz and recorded during the track running session and during resting in a box. For each filtered LFP trace, its standard deviation (STD) was computed and a threshold of 6 STDs was used to detect ripple events (Csicsvari et al., 2000). Ripple start and end times were determined as the time points crossing a threshold of 2.5 STDs. Events with a gap <30 ms were combined to a single event. Only events with durations within [30, 500] ms were considered as ripple events. We quantified each ripple with its peak amplitude, duration, frequency (number of cycles per s). Among all detected ripples, we randomly downsampled them to 6 ripples per animal per session (track running session or resting session). We then compared the ripple parameters between mice and rats for these downsampled ripples. In addition, we computed the average value of each ripple parameter among all detected ripples in a session in an animal, as well as an occurrence rate (number of ripple events *per se*). We also compared these average values of individual animals between mice and rats.

#### Bayesian decoding

We used Bayesian decoding to quantify the accuracy of population spatial coding of the novel track. First, we used the average firing rate curves of all cells active on a trajectory as a template. However, in order to compare the spatial coding between rats and mice fairly, we only used those templates with at least 12 cells, and for those with too many cells we randomly removed some of the cells to make the cell number below 21. In the end, 6 templates were obtained from 3 mice and 6 templates from 3 rats. We then divided the active running time periods of the first 6 laps on a trajectory into 100-ms time bins. The spikes within each time bin were used to decode the probability distribution at each possible position of the trajectory, following a standard Bayesian decoding scheme (Zhang et al., 1998). The position with the peak probability was considered the decoded position at the time bin. The decoding error for the time bin was the absolute difference between the decoded position and the animal's actual position at the time bin. The median error of all time bins in a lap was used as the decoding error for the lap.

#### Theta sequences and replay sequences

We applied the same Bayesian decoding method at small time bins (10 ms) to quantify theta and replay sequences (Johnson and Redish, 2007; Davidson et al., 2009; Karlsson and Frank, 2009). The idea is that if a firing sequence of multiple place cells in a time window (a theta cycle or a ripple event) matches the cells' behavioral sequence on a trajectory, the decoded positions within the window (from the trajectory's template) should match with a portion of the trajectory. For theta sequences, we identified those theta cycles (peak to peak times) with at least 4 active template cells as candidate cycles. For each candidate cycle, we divided it into 10-ms bins. We obtained the decoded position at each time bin by Bayesian decoding and then computed a correlation between the decoded positions and bin numbers of all time bins within a cycle. We then randomly shuffled the time bins 1,000 times and re-computed the correlation. We considered a candidate cycle with its correlation value >95% of the shuffled correlation values as a match cycle. Match ratio, which measured the overall quality of theta sequences given our templates, was the number of match cycles over the total number of candidate cycles. Match ratio was computed for each animal separately and for all mice or all rats combined. Replay events were determined similarly. However, since spikes in a ripple could replay one of the two template, we considered a candidate ripple where its decoded positions matched a portion of a trajectory based on any of the two templates on the track as a replay event (Karlsson and Frank, 2009). Replay ratio was the number of ripples over all candidate ripples before the end of the 6th lap on the track or during the first 20 min of resting. The replay ratio was computed for each animal and for all mice or all rats combined.

## Author contributions

XM, JC, YY, and SK collected and analyzed the data. DJ conceived the project and analyzed the data. All authors wrote this paper.

### Conflict of interest statement

The authors declare that the research was conducted in the absence of any commercial or financial relationships that could be construed as a potential conflict of interest.
